# The triglyceride-glucose index can indicate lipid and atherogenic alterations in adolescents, regardless of excess weight

**DOI:** 10.1590/1984-0462/2026/44/2025215

**Published:** 2026-05-08

**Authors:** Monique de Freitas Mota, Carla Soraya Costa Maia, Géssica de Souza Martins, Thaynan dos Santos Dias, Luis Felipe Nunes de Oliveira, Antônio Ciro de Holanda, Ribanna Aparecida Marques Braga, Eric Wenda Ribeiro Lourenço, Lara Pereira Saraiva Leão Borges, Maria Dinara de Araújo Nogueira

**Affiliations:** a Universidade Estadual do Ceará, Fortaleza, CE, Brazil.; b Universidade de São Paulo, São Paulo, SP, Brazil.

**Keywords:** Adolescent, Biomarkers, Cardiovascular disease, Metabolic syndrome, Obesity, Adolescente, Biomarcadores, Doenças cardiovasculares, Síndrome metabólica, Obesidade

## Abstract

**Objective:**

The aim of this study was to evaluate the role of the triglyceride-glucose (TyG) index in identifying changes in lipid profile and atherogenic risk in adolescents, according to the presence or absence of excess weight.

**Methods:**

This cross-sectional study included 823 students aged 10–18 years, classified as having or not having excess weight based on body mass index. Lipid profile was assessed and classified according to the guidelines of the Brazilian Society of Cardiology. Atherogenic risk was evaluated using the Castelli I and II indices, the Atherogenic Index of Plasma, and the atherogenic coefficient. The TyG index was assessed in tertiles. The association between TyG and lipid and atherogenic outcomes was analyzed using logistic and linear regression, stratified by excess weight status.

**Results:**

Elevated TyG was associated with altered total cholesterol (TC), high-density lipoproteincholesterol (HDL-c), low-density lipoprotein-cholesterol (LDL-c), and non-HDL-c in adolescents without excess weight (p<0.001), whereas in those with excess weight, this association was found only in TC and non-HDL-c. All atherogenic indices were positively associated with elevated TyG (p<0.001).

**Conclusions:**

Adolescents without excess weight showed clearer associations between TyG and lipid profile; however, atherogenic risk was directly associated with elevated TyG, regardless of the presence or absence of excess weight.

## RESUMO

**Objetivo:**

Avaliar o papel do índice triglicerídeo-glicose (TyG) na identificação de alterações do perfil lipídico e risco aterogênico em adolescentes, conforme a presença ou ausência de excesso de peso.

**Métodos:**

Este estudo transversal incluiu 823 estudantes de 10 a 18 anos, classificados com ousem excesso de peso segundo o Índice de Massa Corporal (IMC). O perfil lipídico foi avaliado e classificado conforme as diretrizes da Sociedade Brasileira de Cardiologia. O risco aterogênico foi calculado pelos índices de Castelli I e II, pelo índice aterogênico plasmático e pelo coeficienteaterogênico. O índice TyG foi analisado em tercis. A associação entre TyG e desfechos lipídicos e aterogênicos foi avaliada por regressão logística e linear, estratificada pela presença ou ausência de excesso de peso.

**Resultados:**

TyG elevado associou-se a alterações no Colesterol Total (CT), HDL-c, LDL-c e não-HDL-c em adolescentes sem excesso de peso (p < 0,001), enquanto naqueles com excesso de peso houve associação com CT e não-HDL-c. Todos os índices aterogênicos correlacionaram-se positivamente com TyG elevado (p < 0,001).

**Conclusões:**

Adolescentes sem excesso de peso apresentaram associações mais evidentes entre TyG e o perfil lipídico; contudo, o risco aterogênico relacionou-se diretamente ao TyG elevado, independentemente da presença de excesso de peso.

## INTRODUCTION

 Adolescence is characterized by a transition period during which physiological and behavioral changes lead to increased body adiposity. The puberty process, associated with a more sedentary lifestyle and nutritionally inadequate diets, may contribute to the development of obesity.^1^ In Brazil, data from the Study of Cardiovascular Risks in Adolescents (ERICA) indicated that 25% of adolescents were of excess weight.^2^

 Obesity may contribute to the development of metabolic alterations, such as insulin resistance (IR) and chronic inflammation, which are linked to type 2 diabetes, dyslipidemia, and metabolic syndrome. In obese individuals, dyslipidemia manifests through alterations in lipid profile, including elevated triglycerides, total cholesterol (TC), low-density lipoprotein-cholesterol (LDL-c), and reduced high-density lipoprotein-cholesterol (HDL-c), increasing cardiovascular risk, observed in 60–70% of cases.^3^ Triglyceride-glucose (TyG) index, developed by Simental Mendía,^4^ represents a viable alternative to Homeostatic Model Assessment for Insulin Resistance (HOMA-IR) in clinical practice, with studies confirming its effectiveness for predicting IR in pediatric populations.^5^

 In pediatric populations, assessing IR is more challenging due to physiological changes, especially puberty, which temporarily increases IR. For this reason, there is no consensus on the normal range of HOMA-IR, and TyG has been suggested as a predictor of cardiometabolic risk in adolescents. However, studies in Brazilian populations are lacking.^5^ Studies have shown an association between high TyG levels and lipid profile alterations in children and adolescents, linking them to excess weight and both total and central body fat.^6^ Additionally, other studies associate elevated TyG levels with cardiovascular risks, with a stronger connection when obesity is present.^7^

 Although excess weight is recognized as a factor that can raise TyG levels^8^ and alter lipid profile,^5^ the relationship between this index and atherogenic markers in adolescents without excess weight remains underexplored. This highlights the importance of investigating TyG as a potential marker for early detection of cardiometabolic risk in individuals who do not show changes in body weight but have altered lipid profiles. 

 Thus, considering growing interest in TyG as a marker of cardiometabolic risk,^9^ and the scarcity of studies that stratify findings according to nutritional status, this research aims to evaluate the role of TyG in identifying changes in lipid profile and atherogenic risk in adolescents, according to the presence or absence of excess weight. 

## METHOD

 This cross-sectional study used secondary data from the Dyslipidemia and Dietary Practices in Adolescents in Fortaleza study, collected between April and December 2015. It was approved by the ethics committee of Albert Sabin Children’s Hospital (approval number 327.850) and followed Resolution 466/12. 

 This study population consisted of 65,415 students aged 10–19 years, enrolled in public schools in 2015. Using a formula for finite populations, the sample size was calculated considering a 95% confidence level, a 3% margin of error, and a 5% significance level. A 15% prevalence of dyslipidemia^10^ was assumed for individuals within this age group, resulting in a minimum sample of 775 adolescents. Additionally, a 20% margin was added to account for potential losses, leading to the selection of a total of 930 adolescents. Participants were selected through proportional stratified sampling from 11 public schools in Fortaleza. 

 Stratification was based on the city’s administrative division into six executive regional secretariats (ERS), based on the following criteria:

- Inclusion of schools with highest number of enrolled students;
- Proportional selection of students according to their population in the 10-to-19-year-old age group; and
- Selection through a draw among the students of each class, based on class attendance records and including only those who were present in the classroom on the day of data collection.

Students were distributed across ERS as follows: ERS I – 75 students, ERS II –114 students, ERS III – 82 students, ERS IV – 88 students, ERS V – 234 students, and ERS VI – 337 students. 

 The study included adolescents who signed informed consent forms and provided forms signed by their guardians. The exclusion criteria applied to: participants who were not fasting (12–14 h), pregnant or breastfeeding women, and individuals with physical or mental conditions that prevented participation. Data were collected from 853 adolescents. Participants who presented inconsistencies (missing data or discrepant values) in biochemical and/or anthropometric information in the database were excluded, resulting in a final sample of 823 adolescents.^11^

 Height was measured using a portable rigid stadiometer (Sanny^®^ brand), with the subject standing straight, heels and head touching the stadiometer, and barefoot. Weight was measured using a calibrated portable electronic scale (Seca ^®^ brand). Body mass index (BMI)-for-age was calculated and classified into z-scores based on WHO growth curves.^12^ The following cutoff points were used: underweight (Z-score <-2), normal weight (Z-score ≥-2 and ≤+1), overweight (Z-score >+1 and ≤+2), and obesity (Z-score >+2). For analysis purposes, nutritional status was further categorized into two groups: absence of excess weight (underweight and normal weight) and presence of excess weight (overweight and obesity). 

 Blood samples were collected after 12–14 h of fasting. A 5 mL sample was collected in an EDTA tube for further analysis. In the laboratory, samples were centrifuged at 3000 rpm at 4^°^C for 15 min to separate plasma. Standard methods and commercial kits (Roche Diagnostics^®^) were applied to automated systems (Roche Diagnostics^®^) at the Clinical Analysis Laboratory of Santa Casa de Misericórdia in Fortaleza, Ceará, Brazil. Lipid profile components, such as TC, TG, HDL-c, and glucose, were analyzed. LDL-c was calculated using the Friedewald equation, and non-HDL-c was obtained using the formula (TC−HDL-c). 

 Reference values used to classify lipid profile followed guidelines of the Brazilian Society of Cardiology^13^ for children and adolescents, with the following fasting values considered adequate: TC <170 mg/dL; HDL-c >45 mg/dL; TGs <90 mg/dL, LDL-c <110 mg/dL, and non-HDL-c <120 mg/dL. 

 Atherogenic profile was assessed using Castelli I and II indices, obtained by calculating the TC/HDL-c and LDL-c/HDL-c ratios, respectively.^14^ Atherogenic Index of Plasma (AIP) was calculated as Log10(TG/HDL-c),^15^ and atherogenic coefficient (AC) was determined as (non-HDL-c)/HDL-c.^16^

 TyG was calculated using the following formula: Ln[fasting TGs (mg/dL)×fasting plasma glucose (mg/dL)/2].^4^ For analysis purposes, TyG was divided into tertiles, with the third tertile considered a high TyG (independent variable), as no cutoff points have been published in guidelines or consensus for pediatric populations. This approach was chosen because there are no established cutoff points for TyG in pediatric populations, and dividing variables into tertiles provides a practical and robust way to categorize TyG levels based on their distribution within the sample. Using tertiles allows for clear differentiation of risk levels without relying on arbitrary or unvalidated cutoff points, ensuring the applicability of the results to the studied population. 

 Initially, normality and homogeneity of numerical variables were verified using Kolmogorov-Smirnov and Levene tests, respectively. According to the data distribution, differences between means were evaluated using Student’s t-test for independent samples or the Mann-Whitney U test for dichotomous variables, and analysis of variance (ANOVA) or the Kruskal-Wallis test with Bonferroni post hoc test for polytomous variables. Differences in distributions of categorical variables were tested using Pearson’s χ^2^ test. 

 To assess the association between the independent variable (low TyG vs. high TyG) and the alterations in each component of lipid profile (dependent variables), simple logistic regression (unadjusted model) and multiple logistic regression (model adjusted for sex and age) were performed. Odds ratios (ORs) and their respective 95% confidence intervals (CIs) were presented. Logistic regression with TGs was not feasible, as observed counts in TyG tertile one vs. elevated TG were less than five (zero), violating one of the analysis assumptions. 

 Simple and multiple linear regressions were subsequently conducted to estimate β coefficients and their respective 95% CIs for atherogenic indices (dependent variables) according to the independent variable (low TyG vs. high TyG) in two models: (1) unadjusted and (2) adjusted for sex and age. Dependent variables that did not meet the normality assumption were logarithmically transformed before analysis. TGs and glucose were not included in multivariate analyses due to a strong expected association between these variables and the TyG index, which could lead to collinearity issues. Including these variables in the model could lead to redundant information, compromise parameter estimate accuracy, and hinder the interpretation of independent effects. Therefore, to avoid distorting the results and ensure robust analysis, it was decided not to include TGs and glucose as independent variables in the model. 

 All analyses used Statistical Package for Social Sciences (SPSS) version 22.0. Statistical significance was set at p<0.05. 

## RESULTS

 The study analyzed 823 students, with the majority being female (56.6%) and an average age of 13.08 years (standard deviation [SD]: 1.35 years). Notably, 25.4% of adolescents were overweight. 

 All variables showed significant differences regarding the absence or presence of excess weight, except for TC, LDL-c, and glycemia. Lipid alterations, higher atherogenic indices, and elevated TyG were more frequent in adolescents with excess weight (Table 1). 

**Table 1.** Biochemical data, lipid profile, atherogenic indices, and TyG index in adolescents based on the absence or presence of excess weight (n=823).

Variables		Excess weight		p-value
		Absence n=614	Presence n=209	
TC, mg/dL, mean (sd)		146.71 (27.91)	149.44 (29.31)	0.124^*^
Elevated TC, mg/dL, n (%)		92 (15.0)	40 (19.1)	0.160^†^
LDL-c, mg/dL, mean (sd)		84.21 (21.58)	87.17 (23.78)	0.085^*^
Elevated LDL-c, mg/dL, n (%)		61 (9.9)	27 (12.9)	0.228^†^
HDL-c, mg/dL, mean (sd)		46.54 (10.50)	45.09 (32.51)	<0.001^*^
Low HDL-c, mg/dL, n (%)		272 (44.3)	126 (60.3)	**<0.001^†^**
TG, mg/dL, mean (sd)		78.07 (34.29)	101.74 (62.49)	<0.001^*^
Elevated TG, mg/dL, n (%)		171 (27.9)	98 (46.9)	**<0.001^†^**
Non-HDL-c, mg/dL, mean (sd)		100.17 (26.90)	104.34 (42.44)	<0.001^*^
Elevated non-HDL-c, mg/dL, n (%)		117 (19.1)	62 (29.7)	**0.001^†^**
Glycemia, mg/dL, mean (sd)		84.80 (9.05)	85.47 (9.68)	0.482
Atherogenic indices				
	Castelli I Index, mean (sd)	3.29 (0.91)	3.63 (0.97)	**<0.001^‡^**
	Castelli II Index, mean (sd)	1.91 (0.72)	2.14 (0.75)	**<0.001^‡^**
	Atherogenic Index of Plasma, mean (sd)	0.20 (0.22)	0.33 (0.28)	<0.001^*^
	Atherogenic coefficient, mean (sd)	2.29 (0.91)	2.63 (0.97)	**<0.001^‡^**
	TyG, mean (sd)	8.02 (0.42)	8.24 (0.51)	<0.001^*^
	Low TyG, n (%)	228 (37.1)	50 (23.9)
	Intermediate TyG, n (%)	210 (34.2)	66 (31.6)	**<0.001^†^**
	High TyG, n (%)	176 (28.7)	93 (44.5)

sd: standard deviation; n: frequency; %: percentage; TC: total cholesterol; LDL-c: low-density lipoprotein-cholesterol; HDL-c: high-density lipoproteincholesterol; TG: triglyceride; TyG index: triglyceride-glucose index; Low TyG: <7.85; Intermediate TyG: ≥7.85 to <8.26; High TyG: ≥8.26; p-value:

* Mann-Whitney;

† Pearson’s χ^2^ test

‡ Student’s t-test for independent samples.

Statistical significance: p<0.05. Statistically significant values are denoted in bold.

 By evaluating the distribution of variables by TyG tertiles, it was identified that in the group without excess weight, adolescents with the highest TyG tertile had higher frequencies of alterations in all lipid fractions evaluated and higher atherogenic indices compared to low and intermediate tertiles. Adolescents with excess weight in the highest TyG tertile had higher frequencies of elevated TC, TG, and non-HDL and higher atherogenic indices compared to those with low TyG (Table 2). 

**Table 2.** Lipid profile alterations and atherogenic indices according to TyG tertiles, stratified by the absence or presence of excess weight (n=823).

Variables			TyG tertiles			p-value
Absence of excess weight						
	Lipid profile					
		Elevated TC, n (%)	15 (6.6)	30 (14.3)	47 (26.9)	<0.001^*^
		Elevated LDL-c, n (%)	11 (4.8)	19 (9.0)	31 (17.6)	<0.001^*^
		Low HDL-c, n (%)	85 (37.3)	85 (40.5)	102 (58.0)	<0.001^*^
		Elevated TG, n (%)	0 (0.0)	18 (8.6)	153 (86.9)	<0.001^*^
		Elevated non-HDL-c, n (%)	14 (6.1)	34 (16.2)	69 (39.2)	0.001^*^
	Atherogenic indices					
		Castelli I Index, mean (sd)	2.92 (0.73)^a^	3.20 (0.70)^b^	3.87 (1.05)^c^	**<0.001^†^**
		Castelli II Index, mean (sd)	1.67 (0.53)^a^	1.89 (0.63)^b^	2.27 (0.87)^c^	**<0.001^†^**
		Atherogenic Index of Plasma, mean (sd)	0.01 (0.15)^a^	0.20 (0.11)^b^	0.44 (0.17)^c^	**<0.001^†^**
		Atherogenic coefficient, mean (sd)	1.92 (0.73)^a^	2.20 (0.70)^b^	2.87 (1.04)^c^	**<0.001^†^**
Presence of excess weight						
	Lipid profile					
		Elevated TC, n (%)	4 (8.0)	8 (12.1)	28 (30.1)	0.001^*^
		Elevated LDL-c, n (%)	4 (8.0)	8 (12.1)	15 (16.1)	0.374^*^
		Low HDL-c, n (%)	25 (50.0)	40 (60.6)	61 (65.6)	0.191^*^
		Elevated TG, n (%)	0 (0.0)	10 (15.2)	88 (94.6)	<0.001^*^
		Elevated non-HDL-c, n (%)	7 (14.0)	13 (19.7)	42 (45.2)	0.001^*^
	Atherogenic indices					
		Castelli I Index, mean (sd)	3.01 (0.91)^a^	3.60 (0.72)^ab^	3.99 (0.99)^b^	**<0.001^‡^**
		Castelli II Index, mean (sd)	1.79 (0.75)^a^	2.21 (0.64)^ab^	2.29 (0.77)^b^	**<0.001^‡^**
		Atherogenic Index of Plasma, mean (sd)	0.02 (0.23)^a^	0.27 (0.13)^b^	0.53 (0.20)^c^	**<0.001^‡^**
		Atherogenic coefficient, mean (sd)	2.01 (0.91)^a^	2.60 (0.72)^ab^	2.99 (1.00)^b^	**<0.001^‡^**

sd: standard deviation; n: frequency; %: percentage; TC: total cholesterol; LDL-c: low-density lipoprotein-cholesterol; HDL-c: high-density lipoproteincholesterol; TG: triglyceride; TyG index: triglyceride-glucose index;

* Pearson’s χ^2^

† ANOVA for independent samples;

‡ Kruskal-Wallis with Bonferroni post hoc test

abc different letters indicate differences between groups

Statistical significance: p<0.05. Statistically significant values are denoted in bold.

 Multiple logistic regression showed that TyG was associated with lipid profile alterations in all variables evaluated in the group without excess weight. Adolescents without excess weight but with elevated TyG had higher odds of lipid profile alterations, even after adjustment, compared with those with low TyG. In the excess weight group, associations were found only for TC and non-HDL-c, showing a higher likelihood of alterations in these lipid fractions when elevated TyG was compared with low TyG (Table 3). 

**Table 3.** Association^*^ between elevated TyG index (independent variable) and lipid profile alterations (dependent variables) comparing the highest tertile versus the lowest tertile of TyG, stratified by the absence or presence of excess weight (n=404).

Lipid profile			Excess weight			
			Absence		Presence	
			Low TyG index <7.85	High TyG index ≥8.26	Low TyG index <7.85	High TyG index ≥8.26
Elevated TC						
	Model 1					
		OR (95%CI)	Ref.	5.21 (2.80–9.70)	Ref.	4.95 (1.63–15.09)
		p-value		**<0.001**		**0.005**
	Model 2					
		OR (95%CI)	Ref.	5.19 (2.78–9.70)	Ref.	4.64 (1.51–14.26)
		p-value		**<0.001**		**0.007**
Elevated LDL-c						
	Model 1					
		OR (95%CI)	Ref.	4.22 (2.05–8.66)	Ref.	2.21 (0.69–7.07)
		p-value		**<0.001**		0.180
	Model 2					
		OR (95%CI)	Ref.	4.24 (2.06–8.73)	Ref.	2.17 (0.67–7.02)
		p-value		**<0.001**		0.198
Low HDL-c						
	Model 1					
		OR (95%CI)	Ref.	2.32 (1.55–3.47)	Ref.	1.91 (0.95–3.84)
		p-value		**<0.001**		0.071
	Model 2					
		OR (95%CI)	Ref.	2.35 (1.57–3.53)	Ref.	1.99 (0.98–4.06)
		p-value		**<0.001**		0.058
Elevated Non-HDL-c						
	Model 1					
		OR (95%CI)	Ref.	9.86 (5.31–18.32)	Ref.	5.06 (2.06–12.41)
		p-value		**<0.001**		**<0.001**
	Model 2					
		OR (95%CI)	Ref.	9.68 (5.20–18.01)	Ref.	5.18 (2.09–12.80)
		p-value		**<0.001**		**<0.001**

OR: odds ratio; CI: confidence interval; TC: total cholesterol; LDL-c: low-density lipoprotein-cholesterol; HDL-c: high-density lipoprotein-cholesterol; TyG index: triglyceride-glucose index.

* Multiple logistic regression in two models: (1) unadjusted; (2) adjusted by sex and age.

Statistical significance: p<0.05. Statistically significant values are denoted in bold.

 In linear regression, all atherogenic indices were positively associated with elevated TyG, and this association was significant in both adolescents with and without excess weight (Table 4). 

**Table 4.** Association^*^ between elevated TyG index (independent variable) and atherogenic indices (dependent variables) comparing the highest tertile versus the lowest tertile of TyG, stratified by the absence or presence of excess weight (n=404).

Atherogenic indices			Excess weight			
			Absence		Presence	
			Low TyG index <7.85	High TyG index ≥8.26	Low TyG index <7.85	High TyG index ≥8.26
Castelli I Index^†^						
	Model 1					
		β (95%CI)	Ref.	0.12 (0.10–0.14)	Ref.	0.99 (0.68–1.30)
		p-value		**<0.001**		**<0.001**
	Model 2					
		β (95%CI)	Ref.	0.12 (0.10–0.14)	Ref.	0.99 (0.67–1.30)
		p-value		**<0.001**		**<0.001**
Castelli II Index^†^						
	Model 1					
		β (95%CI)	Ref.	0.13 (0.10–0.16)	Ref.	0.50 (0.25–0.75)
		p-value		**<0.001**		**<0.001**
	Model 2					
		β (95%CI)	Ref.	0.13 (0.10–0.15)	Ref.	0.50 (0.25–0.76)
		p-value		**<0.001**		**<0.001**
Atherogenic Index of Plasma						
	Model 1					
		β (95%CI)	Ref.	0.43 (0.39–0.46)	Ref.	0.51 (0.45–0.58)
		p-value		**<0.001**		**<0.001**
	Model 2					
		β (95%CI)	Ref.	0.42 (0.39–0.45)	Ref.	0.52 (0.45–0.58)
		p-value		**<0.001**		**<0.001**
Atherogenic coefficient^†^						
	Model 1					
		β (95%CI)	Ref.	0.17 (0.15–0.20)	Ref.	0.99 (0.68–1.30)
		p-value		**<0.001**		**<0.001**
	Model 2					
		β (95%CI)	Ref.	0.17 (0.15–0.20)	Ref.	0.99 (0.67–1.30)
		p-value		**<0.001**		**<0.001**

β: beta coefficient; CI: confidence interval.

* Multiple linear regression in two models: (1) unadjusted; (2) adjusted for sex and age;

† Log-transformed variables in the "without excess weight" group;

TyG index: triglyceride-glucose index. Statistical significance: p<0.05. Statistically significant values are denoted in bold.

## DISCUSSION

 Our results showed that TyG is significantly associated with lipid alterations and an increased atherogenic risk in adolescents, regardless of nutritional status. The group without excess weight but with elevated TyG was associated with all evaluated lipid fractions. In contrast, the association was observed only with TC and non-HDL-c in a group with excess weight. All atherogenic indices were positively associated with elevated TyG in both groups. 

 The TyG index was initially proposed as a marker of IR in adults and has proven to be clinically relevant among adolescents, in whom pubertal changes can temporarily increase IR. This evidence supports findings of a study that associates a progressive increase in TyG levels with a higher cardiometabolic risk in adulthood.^17^ In our study, elevated TyG in adolescents without excess weight was associated with a higher chance of alterations in all lipid parameters. Our findings reinforce other studies that evaluated normal-weight adolescents who presented metabolic alterations such as higher android fat, lipid alterations, and TyG values.^18^

 Eutrophic individuals may have increased cardiovascular risk due to a higher percentage of body fat; this phenotype is known as normal weight obesity (NWO). This concept, still underexplored in adolescents, may help explain part of our findings. Adolescents with NWO exhibit higher anthropometric measurements, alterations in cardiometabolic risk markers, greater sedentary behavior, and lower physical fitness than those without. 

 In adults, NWO is associated with hypertension, dyslipidemia, and reduced HDL, but studies in adolescents are still lacking.^18 ,19^

 Our findings suggest that TyG may be a relevant marker for identifying lipid alterations in adolescents with normal weight, even those with a high percentage of body fat. TyG is more effective in the early identification of metabolic changes and IR in NWO adolescents, before the effects of obesity become evident. The lower effectiveness of the TyG index in adolescents with excess weight can be explained by metabolic imbalance from body fat accumulation, due to increased release of cytokines such as interleukin-6 (IL-6) and tumor necrosis factor-alpha (TNF-α), which promote a chronic pro-inflammatory state and an increase in lipid fractions rich in TGs (very low-density lipoprotein [VLDL] and intermediate-density lipoprotein [IDL]) and dysfunction of HDL-c, justifying the lack of association of the index with high LDL and low HDL-c.^20^

 In both groups of adolescents, the association between high TyG and increased TC and non-HDL-c levels can be explained by an increase in VLDL and IDL fractions, in response to higher concentrations of serum TGs. As the index equation includes TGs, their increase directly impacts the rise in TyG. However, metabolic imbalance caused by excess weight can potentiate an increase in serum TGs by intensifying the lipolysis process and reducing the beta-oxidation capacity of fatty acids.^21^

 Inflammatory cytokines resulting from excess weight can alter the enzymatic activity of the lipoprotein axis, such as lipoprotein lipase, contributing to an increase in VLDL and IDL fractions.^22^ This effect directly impacts TC and non-HDL-c biomarkers, reducing LDL conversion and compromising HDL function, which may explain the lack of association of TyG with high LDL-c and low HDL-c levels. We assume these processes are attenuated in adolescents without excess weight, preserving the functionality of the lipoprotein axis, which justifies the association of the TyG index with all lipid profile factors assessed.^21^

 Another point to consider is the size and density of lipoproteins, which were not assessed in our study but are strongly related to cardiometabolic disorders, since individuals with cardiovascular disease have normal LDL-c concentrations but still exhibit small, dense LDL particles, which significantly increase cardiovascular risk due to elevated levels of oxidized LDL (LDLox).^23^ HDL can also undergo qualitative changes, where both its size and cholesterol transport capacity can be affected by metabolic imbalance of excess weight, increasing cardiovascular risk without affecting HDL-c concentrations.^20^

 Studies indicate adolescents with obesity have higher levels of LDLox.^24,25^ This suggests that adolescents with excess weight in our study, even without changes in LDL-c concentrations, may be predisposed to elevated LDLox levels. Considering that the TyG index shows a stronger correlation with LDL particle size than with absolute LDL levels, this association may indicate a more atherogenic pattern.^24^ Regarding oxidized HDL (HDLox), more studies are needed in adolescents with obesity to confirm a similar association. 

 All atherogenic risk indices evaluated in our study were positively associated with elevated TyG, regardless of the presence or absence of excess weight. The literature reports Castelli I and II and AIP indices as efficient markers for identifying cardiovascular risk in adolescents with excess weight. These indices are considered good predictors of metabolic syndrome.^26^ However, when it comes to normal-weight adolescents, there are not enough studies to confirm these claims, which reinforces the relevance of our findings to fill this gap in the literature. 

 Additionally, no studies have verified the association of TyG with atherogenic indices in adolescents, regardless of nutritional status. However, TyG may be a good predictor of subclinical atherosclerosis in young adults,^27^ which may help explain associations found in our study, as altered values of these indices may be precursors of cardiovascular disease in the long term.^28^ Atherogenic indices are more robust predictors of metabolic alterations than isolated lipid parameters, as they integrate atherogenic lipoproteins such as TC, LDL-c, and TG, as well as protective ones like HDL-c.^29^ This approach enhances their association with cardiovascular diseases, reinforcing the importance of incorporating them into the early assessment of adolescents. 

 Although the magnitude of association between TyG and atherogenic indices was slightly greater in the group with excess weight, this effect likely reflects the cumulative impact of IR and adiposity. Notably, most consistent associations with individual lipid parameters were observed among adolescents without excess weight. This finding suggests underlying mechanisms may vary according to metabolic profile, without compromising the utility of TyG as a versatile marker of cardiometabolic risk across different populations.^30^

 In addition to assessing nutritional status, our results may have been influenced by dietary factors. Cultural aspects of Brazilian eating habits, which are based on a Western diet, are characterized by high consumption of hypercaloric foods, saturated fatty acids, and high-glycemic-index carbohydrates, components consistently associated with cardiovascular disease development. Similarly, behavioral variables such as physical activity levels and sleep duration may influence adolescent metabolic alterations.^31^

 Among the study’s limitations, the cross-sectional design precludes causal inference. Additionally, unmeasured factors such as detailed diet parameters, physical activity, body composition, socioeconomic indicators (income and parental education), screen time, and medical history could influence both adiposity and glucose–lipid metabolism, potentially modifying the TyG index–lipid profile relationship. Absence of these data prevented their inclusion in multiple models. However, in Brazil, public schooling can be considered an indicator of social vulnerability, and adjustments for sex and age help minimize physiological and biological biases among the adolescents assessed. 

 Some strengths should be emphasized, such as representativeness of adolescents within the studied population and use of TyG tertiles, which, despite a lack of consensus regarding cutoff points, allowed its evaluation among adolescents with elevated values, facilitating the identification of its associations with clinical outcomes and encouraging its use in clinical practice and research. To our knowledge, this is the first study to evaluate the relationship between the TyG index and atherogenic indices in adolescents, stratified by the presence and absence of excess weight, highlighting the need for public policies to identify cardiovascular risk factors in normal-weight populations. 

 We conclude that elevated TyG levels were associated with lipid profile alterations and an increased atherogenic risk among adolescents evaluated. Associations between TyG and the lipid profile were more evident in those without excess weight. However, although atherogenic risk was significantly associated with TyG in both groups, the magnitude of this association was stronger among adolescents with excess weight. 

 These findings suggest TyG may serve as a valuable biomarker for early identification of atherogenic risk in adolescents, regardless of nutritional status, aiding preventive strategies and early management of metabolic alterations via an accessible, non-invasive tool. Notably, they highlight the need for longitudinal studies to assess TyG’s predictive role over time and its applicability across pediatric populations, reinforcing its relevance as an early cardiometabolic risk marker. 

## Data Availability

The database that originated the article is available with the corresponding author.
